# Standardising job descriptions in the humanitarian supply chain: A text mining approach for recruitment process

**DOI:** 10.1371/journal.pone.0305961

**Published:** 2024-07-10

**Authors:** Irene Spada, Valeria Fabbroni, Filippo Chiarello, Gualtiero Fantoni

**Affiliations:** 1 Department of Energy, Systems, Land and Construction Engineering, School of Engineering, University of Pisa, Pisa, Italy; 2 Business Engineering for Data Science (B4DS) Research Lab, School of Engineering, University of Pisa, Pisa, Italy; 3 Department of Civilisations and Forms of Knowledge, University of Pisa, Pisa, Italy; 4 SDCC Department, Asian Development Bank, Fragile Countries, Manila, Philippines; 5 Department of Industrial and Civil Engineering, School of Engineering, Università di Pisa, Pisa, Italy; The University of Jordan Faculty of Business: The University of Jordan School of Business, JORDAN

## Abstract

**Purpose:**

Uncertainty and complexity have increased in recent decades, posing new challenges to humanitarian organisations. This study investigates whether using standard terminology in Human Resource Management processes can support the Humanitarian supply chain in attracting and maintaining highly skilled operators.

**Methodology:**

We exploit text mining to compare job vacancies on ReliefWeb, the reference platform for humanitarian job seekers, and ESCO, the European Classification of Skills, Competencies, and Occupations. We measure the level of alignment in these two resources, providing quantitative evidence about terminology standardisation in job descriptions for supporting HR operators in the Humanitarian field.

**Findings:**

The most in-demand skills, besides languages, relate to resource management and economics and finance for capital management. Our results show that job vacancies for managerial and financial profiles are relatively more in line with the European database than those for technical profiles. However, the peculiarities of the humanitarian sector and the lack of standardisation are still a barrier to achieving the desired level of coherence with humanitarian policies.

## 1. Introduction

Humanitarian supply chain management is gaining more and more attention due to the increased number of disasters, both man-made and natural [1]. Those entail many global issues, such as climate change and the COVID-19 pandemic, the recrudescence of wars, such as the Afghan debacle or the recent Russian-Ukraine and Israel-Hamas conflicts, as well as recurrent financial crises. Humanitarian organisations should strive to provide more effective interventions in responding to the growing needs [2] and in avoiding adverse impacts in achieving the Sustainable Development Goals (SDGs) in accordance with Agenda 2030 [3]. A suitable approach encompasses leveraging highly skilled operators [4], ensuring strong relationship management capability [5], and providing an efficient utilisation of money [6], as *conditio sine qua non* for a sustainable future of humanitarian supply chains. Moreover, a lack of coordination can lead to inefficient and ineffective humanitarian aid [7]. The humanitarian response includes not only practical assistance addressing the needs of affected people but also a responsibility to manage and coordinate the efforts of different bodies and agencies and provide a single point of contact for donors [8].

The escalating awareness of social and environmental challenges on a global scale has pushed Non-Governmental Organizations (NGOs) into pivotal roles. These entities are increasingly recognised as essential in addressing critical issues, necessitating an entrepreneurial mindset to navigate challenges and opportunities [9]. Moreover, the globalisation of the humanitarian supply chain requires the involvement and active collaboration of many actors and stakeholders of different cultures, leading to the increasing importance of management capabilities [10–12].

However, the key figures in charge of those activities may not always espouse the appropriate competencies and skillsets, leading to poor performances of the system and lessening of public confidence in them [13]. Therefore, all the reasons above have made it increasingly important to have well-trained personnel to manage the supply chain [4]. Whereas the need for progressive professionalisation is strongly felt by the humanitarian community, as proven by the increasing number of procedures and certifications at the organisation level, the humanitarian sector is still anchored to its philanthropic and mission-driven origins, which weakens the foundations for the development of skills and competencies that can help better respond to the volatile environment [14]. A standard architecture of the roles in the humanitarian supply chain can be paramount for the quality of NGOs’ interventions [15]. Human capital is the most important asset in every company, and effective communication of hiring needs within the recruitment process is fundamental [16]. The authors in the literature review [17] highlight the importance of a standard terminology in Human Resources Management (HRM) for properly addressing processes, functions, and required skills. In this sense, the standardisation of the recruitment language can help to attract and evaluate candidates, as a standard language provides a reference for the definition of the requirements of a given position, for the attribution of responsibilities, and the evaluation of performances [18–20].

This paper takes up the research challenges posed by the authors in [4] in their literature review of humanitarian supply chains. The authors highlight the need for studies on human resources management that exploit quantitative methods and aim to analyse HR practices’ contribution to the performance of humanitarian operations. We focus on the utilisation of standard terminology to address the skills needs of humanitarian professions, aiming at answering the following Research Question: *Does the European Humanitarian HR supply chain use standard terminology in its HRM processes*?

We propose a quantitative comparison between the job vacancies available on ReliefWeb (accessible at https://reliefweb.int/), the main source of exchange between possible employers and job seekers in the humanitarian sector, with the European Classification of Skills and Occupations (ESCO, accessible at https://esco.ec.europa.eu/en), the reference framework for the labour market in Europe. We use text mining techniques to measure the level of agreement in the terminology adopted in the two sources mentioned above. This paper presents quantitative insights on humanitarian supply chain skill needs and allows us to frame the requirements for professionals in the field, enabling standardised skill and career models. Our results show that job vacancies for managerial and financial profiles are more in line with the European classification, compared to operational profiles; anyway, it is evident that for all of them there are still gaps in the definition of quality standards to reach the adequate level of coherence with policy documents.

This research is structured as follows. Section 2 gives an overview of the literature related to humanitarian sectors and its main gaps. Section 3 explains the methodology, from the preparation to data elaboration. Section 4 presents the analysis results, summarising and discussing the results. Finally, Section 5, describes the conclusions in response to the questions posed in the present Section 1, including the possible applications and acknowledging some limitations of the proposed approach, which open challenges for future studies.

## 2. Literature review

### 2.1 Post-COVID humanitarian needs overview

According to the report [1] from the Coordination Office of the United Nations in 2021, 235 million people needed humanitarian assistance and protection. This number has risen to 1 in 33 people worldwide, a significant increase from 1 in 45 at the launch of the Global Humanitarian Overview 2020, which was already the highest figure in decades. Until 2019, a slow but steady improvement in food security, the fight against malaria and improvement of living conditions amongst the most vulnerable segments of the worldwide population could be observed; with the spread of the COVID-19 pandemic in 2020, however, a worsening of the general situation is estimated to bring the development path of countries in need back to the conditions of almost 30 years ago [1]. This enormous step back impacts the possibility of achieving the Agenda 2030 targets for 17 parameters of the Sustainable Development Goals (SDGs). The SDGs outline the progress to be made with regard to hunger, education, equality, partnership for development, water and sanitation, health, and ICT [3].

The recent conflicts in Ukraine (2022 –present), Sudan (2023 –present), and Gaza (2023-present) further aggravate the overall situation. In 2022, the Collins Dictionary announced that the term *permacrisis*, defined as *an extended period of instability and insecurity*, *especially one resulting from a series of catastrophic events*, *is the most representative word of the year*.

### 2.2 The role of Non-Governmental Organizations (NGOs) in responding to needs

NGOs’ response to pressing humanitarian needs requires appropriate quality of interventions, both from the humanitarian imperative point of view and accountability towards donors and clients. Considering the first perspective, the State of the Humanitarian System (SOHS) biannual publication [21] highlights the evolution of humanitarian needs and the response by the humanitarian community: it shows an increasing trend in the number of people in need, which presently reaches 201 million, mainly counted in the Syria South Sudan and Yemen crisis. In 2016, the Istanbul World Humanitarian Summit (WHS), resulting from more than a year of regional consultations amongst UN Agencies, civil society organisations, and heads of State and private sector members, brought together 9,000 participants. Those represented 180 Member States, including 55 Heads of State and Government, hundreds of civil society and non-governmental organisations, and partners, including the private sector and academia. Together, they generated more than 3,500 commitments to action. Amongst the others, the WHS aimed to localise aid and the selection of the operators to reduce the imbalance between expats from North Europe and the West and other humanitarian operators coming from the perceived South partnerships and initiatives to turn the Agenda for Humanity into meaningful change for the world’s most vulnerable people [22]. Hence, focusing on donors and clients who finance humanitarian operations, NGOs are called to demonstrate effectiveness and efficiency in relief response [23]. NGOs contribute to humanitarian intervention alongside multiple stakeholders in multifaceted contexts and resource-intensive operations; this requires transparency towards donors and efficiency in using the resources allocated as elements necessary to build cooperation and trust [24,25].

### 2.3 Professionalization of humanitarian operators

The rapid pace of socio-technical transformation in the last decades affects industries and labour markets, calling for new professionals and skills [26,27], including procedures and practices in recruiting NGOs’ staff.

Managerial capabilities and soft skills such as teamwork, ability to listen and communicate [28,29], analytical capabilities regarding the use of ICT, data gathering and exploitation techniques [30,31], as well as the knowledge management processes and procedures to share timely information during crisis [32], strategic ways of thinking, all those are becoming paramount to develop and implement win-win solutions in such fast-moving and unpredictable changing environment. It is now recognised that NGO operators should be able to perform needs analysis for providing proper supplies and resources in relief; they should demonstrate managerial capabilities in planning and coordinating activities, as well as financial planning to manage relations and develop trust along the supply chain [33]. In particular, the Humanitarian Supply Chain includes processes and activities addressing management and coordination in humanitarian aid and relief, collaboration and cooperation between stakeholders, logistics for the movement of goods and resources, and information sharing among suppliers and beneficiaries [34]. The primary objective is to save and restore affected populations by crisis and disasters, pursuing visibility, agility, and the utilisation of information technology to improve performance and response capabilities. Indeed, humanitarian supply chain management as a concept recognises the unique challenges and requirements of delivering aid and timely and appropriate assistance [35].

More than ever, the skills required in the humanitarian supply chain are a mix of transversal, managerial, and technical competencies [36].

In the study [20] on skills for logisticians in the humanitarian context, the authors leverage job vacancies on ReliefWeb to measure the skills demand in this area. They highlight five competence areas, namely *General Management Skills*, *Functional Logistics Skills*, *Interpersonal Skills*, *Problem Solving and Personality Traits*, and finally, *Skills for the Humanitarian Context*, giving evidence of the importance of technical capabilities related to the function (i.e., logistics in the analysed case) and transversal context-specific ones for the peculiarities of the humanitarian operations. In the report [11] on the evolution of Humanitarian needs and the shrinking of humanitarian space in crisis and conflicts, the author highlights how the increasing complexity of the political scenarios and the recrudescence of conflicts in fragile states “requires dedication to political solutions to complex crises and a willingness to engage—bilaterally and multilaterally—in committed, sustained humanitarian diplomacy” [11]. This further layer of complexity brings the level of skills and competencies for humanitarian operators to a higher level of specialisation in negotiation and soft skills. In addition, scholars in [10] argue about the role of governments in disaster relief, which act as facilitators, promoters, and supply network members. Therefore, humanitarian operators must be able to interact in this network, leveraging communication and information-sharing abilities, coordination, collaboration and partnering capabilities [10]. The core competencies to ensure cooperation between humanitarian organisations and defence institutions: information knowledge management, needs assessment tools, logistics capabilities with reference to supply, deployment and distribution, and health support service [12,37]. The cited studies highlight the importance of leveraging, on the one hand, the rapidity and the efficiency of defence bodies and, on the other hand, the deep understanding of assistance dynamics of humanitarian organisations to effectively achieve restoration. In addition, authors in [38] identify, through a Delphi study, the list of content areas for cultural awareness training programs addressing humanitarian staff. Those include cultural awareness sensitivity, intercultural communication, unconscious biases, diversity awareness, empathy, cultural intelligence, gender and LGBTQ issues and safety, universal declaration of ethical principles, power dynamics, negotiation, program mission, and analytical and critical thinking skills. Next, the essay [18], using the job ads of ReliefWeb, scholars discusses the need for a *Climate Science Translator*, a specialised career in the humanitarian field to face the impact of extreme climate events which acts as a broker agent among academics, scientists and practitioners on climate science data and provide support to decision-makers in NGOs. This profile should demonstrate a mix of technical knowledge on climate and risk management and the ability to communicate and proactively interact with humanitarian operators effectively.

### 2.4 The Changing humanitarian human resources

The literature review [4] highlights the need for a restructuring of HR supply chains in the humanitarian sector, considering the peculiarity of the sector in which the motivation of humanitarian workers often goes beyond retribution or career paths, deployment locations, and salary conditions are generally not particularly competitive nor appealing; they also require efforts for adaptation to difficulties and challenging environments. Whereas responding to humanitarian needs requires professionalism and technical skills, authors in [4] highlight how the staff engaged in these jobs are moved by personal and philanthropic reasons. Building on the authors’ observation in [4], it can be stated that, despite the importance of tasks implemented at the service of people in need, the humanitarian sector suffers from a scarce level of professionalisation, attracting a low specialisation level of operators. Indeed, many difficulties of humanitarian organisations in hiring and recruitment are linked to the volunteer base of work: people who work in humanitarian organisations are volunteers, and this can make it difficult to find qualified staff [39], especially for managerial positions [40]. Additionally, employees may change from one organisation to another due to disaster-specific staffing requirements, increasing the turnover rate and difficulty keeping track of qualified candidates [41]. This leads to poor performances in the field, giving way to scandals of mismanagement of funds and underachievement of what supposedly happened with public funds. Poor achievements bring low confidence in public and institutional donors, reducing, *de facto*, the volume of donations and the possibility to contribute to overall objectives as defined by the Sustainable Development Goals (SDG) and Emergencies Humanitarian Response Plans. Similarly, authors in [42] conducted a survey on behalf of Enhancing Learning & Research for Humanitarian Assistance (ELRHA) of over a thousand aid workers to better understand how they perceived their work and notions of professionalism associated with it. Of the respondents to the survey, 92% indicated that they supported notions of professionalising the work and structure of humanitarian aid. Respondents went on to detail the values, skills, and knowledge that they thought aid workers needed, the support structures they should have, and the methods for codifying competence that they thought should be implemented. Five critical areas for advancement were identified: core competencies, certification systems, apprenticeship and experiential learning, professional associations, and accreditation and accountability. The reinforcement of the humanitarian supply chains was generally perceived in terms of skills (such as resilience and cultural awareness) and roles and professions (e.g., managers, doctors, engineers). The reinforcements also pass through more transparent communication of requirements and needs in such labour markets. Indeed, in [43], analyse each of the 5 points, reviewing existing literature and examining the existing bodies of accreditation for the training of qualified and experienced Humanitarian workers, advocating for creating a codified system. The importance of well-established and up-to-date language for expressing skills needs is fundamental to support employers and job seekers in the labour market’s dynamics, ensuring the proper communication of skills demand and offer [44]. In this sense, standards and regulations can support the humanitarian sector with a clear and fast decision-making process in disaster relief [15,17].

### 2.5 Towards the building of a competencies framework

The CHS Alliance published in 2016 the first attempt to support organisations in implementing competency frameworks, the Core Humanitarian Competency Framework (CHCF) [45]; it introduced a competency-based approach across the organisation. The guide also helps organisations conduct competency-based interviews, assess and develop competencies, recruit and retain staff with the necessary competencies for effective humanitarian response, and enhance employee professionalism. Additionally, it establishes a standardised set of skills, behaviours, and abilities required for successful performance in specific jobs or roles within the organisation. The framework provides clarity and guidance on the skills, knowledge, and behaviours necessary to ensure a high-performance standards. Overall, introducing an organisational competency framework helps improve talent management processes, align the workforce with strategic goals, and foster a culture of continuous improvement and development.

In [2], the Bioforce institute, Centre of Excellence for the training and preparation of Humanitarian operators, mentions that “as a general rule, humanitarian workers did not identify with a specific humanitarian profession, but rather with the humanitarian sector in general.” Many professions (such as logistic coordinator or financial manager) have been adapted to the humanitarian sector, while domain-related tasks and activities do not undergo structured job profiles.

Therefore, a stronger standardisation of the description of the jobs can support employment processes in the field (see, for example, the case of the nurses described in [46]). On one hand, for employers in the field, a standard language eases communicating expectations on candidates and requirements for the roles with transparency: clear communication not only allows for attracting more suitable candidates but also reaches a broader audience [47]. On the other hand, when job seekers understand expectations and requirements, they are more likely to apply, leading to a larger pool of qualified candidates for the position; moreover, candidates can better assess their suitability for the role and the responsibilities associated with the position [48]. The importance of guidelines is thus perceived especially for HR operations: a common disaster curriculum with homogeneous definitions in terms of roles and competencies can be a game changer in the field of humanitarian assistance and disaster relief [49–51].

## 3. Data and method

This paper investigates the standardisation of the terminology used in job recruitment operations in the Humanitarian sector. We selected the data and structured the method articulating the Research Question reported in Section 1 in the following hypothesis:

I***f*** *the description of profiles can be grouped for skills and competencies and the profiles can be aligned with ESCO taxonomy*, ***then*** *the definition of the key positions’ selection criteria can be standardised*. ***Therefore*,** *it is possible to set up a consistent method for supporting HRM processes*.

First, we collected the job vacancies from the ReliefWeb portal, adopted by scholars to study the trends in the humanitarian-related labour market [18,20]. Secondly, we analysed the textual content of the job vacancies, leveraging text mining as a reference framework for the European Classification of Skills and Occupations. Text mining is a technique for the automatic extraction of information from written sources. It mixes Natural Language Processing (NLP, the process through which natural language is transformed in structured data), statistics and knowledge management [52]. We adopted the ESCO dataset, which has been used as a reference framework in many previous works (e.g., [44,53,54]). The present study applies a similar approach to the field of humanitarian recruitment. We used string matching algorithms to identify the ESCO skills in the job vacancies and similarity algorithms to measure the alignment of the job descriptions in the ads and the occupations’ descriptions in the ESCO taxonomy. The derived quantitative information allowed us to compare the two sources, aiming at examining alignments and gaps.

### 3.1 Data

#### 3.1.1. Job vacancies

Job vacancies are considered one of the main sources of information that represent the needs of the labour market [55]. For the scope of this analysis, we relied on ReliefWeb, a humanitarian information service provided by the United Nations Office for the Coordination of Humanitarian Affairs (OCHA). It is a platform of humanitarian information exchange commonly used as a reference by the main donors, including ECHO. It is one of the most consulted job repositories for Humanitarian workers, including posts for jobs, consultancies, internships, and volunteer opportunities. Vacancies from the main agencies and NGOs, with snapshots about worldwide crises and information on emerging needs, converge on this website. Job ads are published in English and French. We collected 3,364 job vacancies available online in May 2020 on ReliefWeb through the ReliefWeb API (accessible at https://apidoc.rwlabs.org/#introduction). We selected ads in English and originating from organisations with legal headquarters in Europe to measure the alignment with the European ESCO classification. Out of the collection, 1,457 entries were identified as eligible (1,441 when removing duplicates). The duplicates refer to the same offer applied to various countries of deployment. This is a common case whereas the same NGO, or one part of an umbrella of NGOs (i.e., Save the Children Italy as part of the Save the Children network, Oxfam Italy as Part of the Oxfam federation and so on), publishes a vacancy for a given position in several countries at the same time. Such cases were not considered for the scope of this work.

#### 3.1.2. Skills and occupation

The European Classification Skills, Competences, Qualifications, and Occupations (ESCO) is a multilingual classification developed in 2008 by the European Commission and is constantly updated to align with the evolution of the labour market. It is based on the International Standard Classification of Occupations (ISCO-08). It has been widely used in literature to reference those concepts (as reported and adopted in [44,53,54]). We used version V1.0.8, published in August 2020, to keep the same time window of the available job vacancies. This version includes 13,485 skills and 2,942 occupations. Skills, knowledge, competencies and occupations have been defined by different authors with different interpretations. For authors in [56], “although there is a proliferation of indicators and benchmarks for skills in the EU, […] there is no common and consistent conceptual definition, which makes them difficult to compare, sometimes even contradictory”. In 2014, CEDEFOP, defined skill as “the ability to apply knowledge and use know-how to complete tasks and solve problems”; while competence as “the ability to apply learning outcomes adequately in a defined context (education, work, personal or professional development) and is not limited to cognitive elements, but also encompasses functional aspects and interpersonal attributes and ethical values”. In this sense, competence includes the capacity to act autonomously and with responsibility and act [57]. Those definitions have been confirmed by the OECD [58]. Then, the International Labor Office defined the concept of a job as “a set of tasks and responsibilities performed by one person” and an occupation as “‘a set of jobs whose main tasks and duties are characterised by a high degree of similarity” [59]. The ESCO handbook provides the following definitions for the concepts mentioned above:

- **Knowledge**: “The body of facts, principles, theories and practices related to a field of work or study. Knowledge is described as theoretical and/or factual, and is the outcome of assimilating information through learning” [60].
- **Skill**: “The ability to apply knowledge and use know-how to complete tasks and solve problems. Skills are described as cognitive (involving the use of logical, intuitive and creative thinking) or practical (involving manual dexterity and the use of methods, materials, tools and instruments)” [61].
- **Competence:** “The proven ability to use knowledge, skills and personal, social and/or methodological abilities, in work or study situations, and in professional and personal development” [62].
- **Occupation**: Group of “jobs involving similar tasks, and which require a similar skills set” [63].

The terms “skill” and “competence” refer to the use of knowledge for performing a given task and the ability to apply its own know-how for facing working or studying challenges. However, the ESCO database does not distinguish between skills and competencies, while it distinguishes between skill/competence and knowledge concepts. We will stick with the ESCO definitions in our analysis. Each skill or knowledge concept has a description, a leading label and some alternative labels, and it is related to one or more jobs. Each occupation concept contains a description, a main label, some alternative labels, and a list of related skills and knowledge.

### 3.2 Method

In this section, we describe the method of our analysis, divided into three phases: (1) data preparation, (2) data processing, and (3) data analysis.

#### 3.2.1 Data preparation

As mentioned in 3.1.1, 1,457 (1,441 as unique) job vacancies from ReliefWeb have been selected for this analysis. The unstructured text of the job vacancies was manipulated using standard cleaning procedures. First the text was tokenised into paragraphs, using as a split character the new line. Then, the length of the paragraphs was used to filter not relevant parts:

- Paragraphs with less than three terms were eliminated, being considered as titles of the sections of the job offers.
- Paragraphs with more than 200 terms were eliminated, as they contained information that was not useful for the job description, such as information about the employer, the legal framework under which the vacancy was published, or a description of the context of intervention.

The relevant paragraphs underwent lemmatisation, which reduces a word to its root, which is called a *lemma*.

#### 3.2.2 Data processing for skill extraction: String match algorithm

Skills extraction was executed using the ESCO skills and knowledge as a reference lexicon. Each preferred label and all the alternative labels of the ESCO concepts (i.e., skills and knowledge) were searched with a string match algorithm as a pattern in the raw and lemmatised text of the vacancies from ReliefWeb. The approach implements a combination of functions from the package *Stringr* and *Dplyr* in RStudio [64,65]. This step led to a list of skills and knowledge for each job vacancy. Then, the extracted concepts were manually revised by one of the authors, who had professional experience in humanitarian operations and crisis management, to check the consistency between the job ads and the skills and knowledge automatically associated with them. This revision allows us to measure the performance of the extraction algorithm.

#### 3.2.3 Data processing for occupation’s comparison: Word2vec similarity

Job descriptions from the job vacancies in ReliefWeb were compared to the description of occupations in ESCO using semantic similarity. We computed the cosine similarity between the two elements using Word2vec [66] to represent the word embeddings. Word2vec embeds words into vector space and then quantifies words’ distance by calculating the cosine of each pair of vectors. This step led to a list of pairs consisting of a job title from ReliefWeb and an occupation from ESCO; for each pair, a measure of their similarity is provided.

#### 3.2.4 Data analysis

In line with the scope of this research, the degree of terminology’s standardisation of the NGOs’ job positions in the humanitarian sector was measured by using two indicators:

1. the *Percentage of Shared Skills* by a job vacancy of ReliefWeb and an occupation in ESCO, coming from the skill extraction process, is computed as follows. $Percentage\;of\;Shared\;Skills\;[\%]=\frac{number\;of\;shared\;skills}{number\;of\;skills\;per\;occupation}*100$
  where the *number of shared skills* is the number of equal skills extracted in a job vacancy of ReliefWeb and associated with a given occupation in ESCO, and the *number of skills per occupation* is the number of skills associated with a given occupation in ESCO. We specify that the *number of skills per occupation* considers only the skills and the knowledge associated with a given occupation as “essential”: ESCO uses a tag to differentiate the competencies usually required for an occupation from the optional ones (not so often required).
2. the *Similarity Score* is the cosine similarity between the two elements using Word2vec to represent the word embeddings.

The metrics have two different focuses. The first metric considers *skills* and *knowledge*, comparing the list of ESCO concepts extracted from two sources (i.e., ReliefWeb and ESCO). The second focuses on occupations because it references the whole job offer of ReliefWeb and the entire occupation description of ESCO. These two metrics have been computed for all the possible pair of job offers from ReliefWeb and occupations from ESCO for a total of 3,768,702 pairs (i.e., number of job vacancies multiplied by the number of occupations). It is evident that such a comparison can be performed only with the support of an automatic algorithm and that a critical analysis is necessary to evaluate the results. Indeed, the statistical analysis of the distribution of the two indicators lets us define four classes of results, highlighting alignments and gaps among the two sources. The statistical significance of the numerical results is then tested to verify the analysis’s robustness and relevance.

The two indicators, namely the *Percentage of Shared Skills* and the *Similarity Score*, can thus measure the degree of standardisation of the NGOs’ job positions with ESCO terminology and help standardise job descriptions. In the approach proposed here, if the two indexes are higher, the standard terminology of ESCO and the job vacancies in the field are aligned, providing a kind of validation for the job ads. On the contrary, when both indicators report low values, the need for standardisation emerges, highlighting the absence of a more comprehensive terminology. The alternative positions can suggest a standardisation in the job description (i.e., high *Percentage of Shared Skills* but low *Similarity Score*) or an expansion of the standard terminology (i.e., low *Percentage of Shared Skills* but high *Similarity Score*).

## 4. Results

This section shows the results of the analysis, starting with a summary of the metrics deriving from the preparation and processing phases to examine the four different cases of the degree of standardisation of the NGOs’ job positions. Finally, an in-depth discussion of a selection of entries for each case is reported.

### 4.1 Descriptive metrics

Table 1 reports metrics on skills extracted from the job vacancies using a string match algorithm.

**Table 1:** Metrics on skills extracted from the job vacancies using string match algorithm.

Metrics	Values
Number of total job vacancies (online in May 2020)	3,364
Number of eligible job vacancies	1,457
Number of unique job vacancies	1,441
Total number of sentences in job vacancies	56,395
Percentage of job vacancies in which at least one concept of ESCO has been detected with respect to the number of unique job vacancies	89,5%
Total number of skills extracted	25,336
Total number of different skills extracted	687
Average skills per job vacancies	20.8

The performance of the skills extraction algorithm was evaluated to verify if the identified skills were correct or not within a given job vacancy. We reviewed a random sample of 200 sentences; in particular, 100 are related to the extraction in the raw version of the texts, and 100 are related to the extraction in the lemmatised version. We measured the precision, i.e., the fraction of number of correct extractions with respect to the total number of extractions in the sample considering different perspectives:

- We conducted a general check for each job vacancy, checking whether the full list of identified skills aligned with the job offer.
- We conducted a detailed check for each sentence of each job vacancy, checking whether the extracted skill was coherent with the sentence.

We computed these measures for all the extractions and separately for the extractions from raw text and lemmatised text. The review’s results are reported in Table 2.

**Table 2:** Precision metrics from review results.

Precision Metrics		Sample		
		All extraction	Extraction from raw text	Extraction from lemmatized text
**Type of check**	General	86,00%	91,00%	81,00%
	Detailed	78,28%	57,05%	21,23%

The performance of the extraction algorithm is quite good, as it can identify meaningful information in 86% of the analysed cases. The extraction from raw text, where exactly the sequence of terms has been found in the original text, is more precise than the extraction from lemmatised text. The achieved precision values align with the obtained values in the labour market intelligence stream of literature (e.g., [54,67,68]).

Table 3 reports the 20 most recurrent skills and knowledge detected in the job vacancies. We can observe that most entries (12, highlighted in bold) are classified as knowledge in ESCO, and then we can find two languages (English and French) and six skills. Some concepts refer to the economic and financial knowledge for capital management, like *Securities* and *Economics*, which can be very important in managing the relations with donors who finance the humanitarian operations, especially if considered together with *Politics* (defined in ESCO as “the method, process and study of influencing people, gaining control over a community or society, and the distribution of power within a community and between societies”.) Then, several deal with resources management, such as *Logistics* or *Programme Work According to Incoming Orders*. Many skills rely on collecting, processing and presenting information, such as *Communication* and *Terminology*. Next, we can find some entries related to managerial areas, such as human resource management and project m*anagement*. Finally, two soft skills (leadership and creativity) appear in this top 20.

**Table 3:** Top 20 most recurrent ESCO skills detected in the job vacancies of Reliefweb.

Skills	Percentage of Job Vacancies mentioning the given skill
**Securities**	77,31%
**Communication**	71,34%
**Logistics**	66,90%
English	63,71%
**Statistics**	51,01%
**Journalism**	47,54%
**Terminology**	43,58%
**Politics**	38,86%
**Design Principles**	38,58%
Think Creatively	38,24%
Provide Leadership	37,61%
Design Ventilation Network	37,13%
Perform Ground-Handling Maintenance Procedures	29,77%
Compile Airport Certification Manuals	27,41%
**Human Resource Management**	26,51%
**Economics**	26,30%
French	24,77%
Programme Work According to Incoming Orders	24,01%
**Electricity Principles**	23,53%
**Project Management**	21,58%

### 4.2 Degree of standardisation of the NGOs’ job positions

The degree of standardisation of the NGOs’ job positions can be derived as a combination of the indicators used in our analysis, namely the *Percentage of Shared Skills* and the *Similarity Score*.

The correlation between the two indicators is quite low: the Pearson’s correlation is equal to 0.08 at the 5% significance level, with a p-value of 2.2e-16 [69], meaning that the obtained value of correlation is statistically significant and that there is no relevant relationship between the two variables. Thus, the indicators mean they observe the same phenomena (i.e., terminology standardisation) from two perspectives. As mentioned, the Percentage of Shared Skills measures the use of standard terms in the job offers, considering the ESCO classification of skills as a reference. At the same time, the *Similarity Score* quantifies the standard concepts included in the job vacancies, considering the ESCO occupation classification as a reference. We computed the two indicators on all the possible combinations of job offers from ReliefWeb and occupations from ESCO for a total of 3,768,702 pairs.

**Fig 1:**
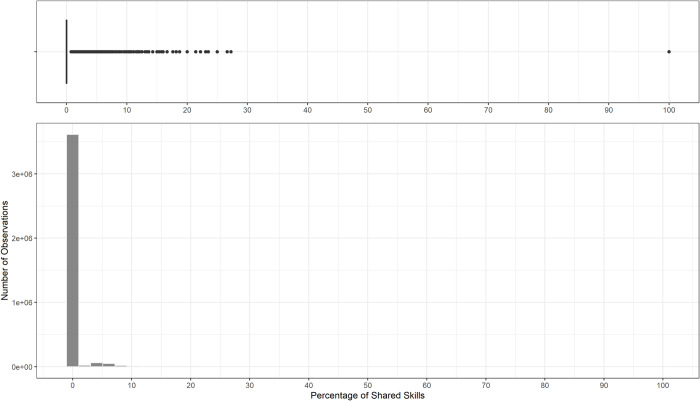
Histogram and boxplot of the percentage of shared skills.

**Fig 2:**
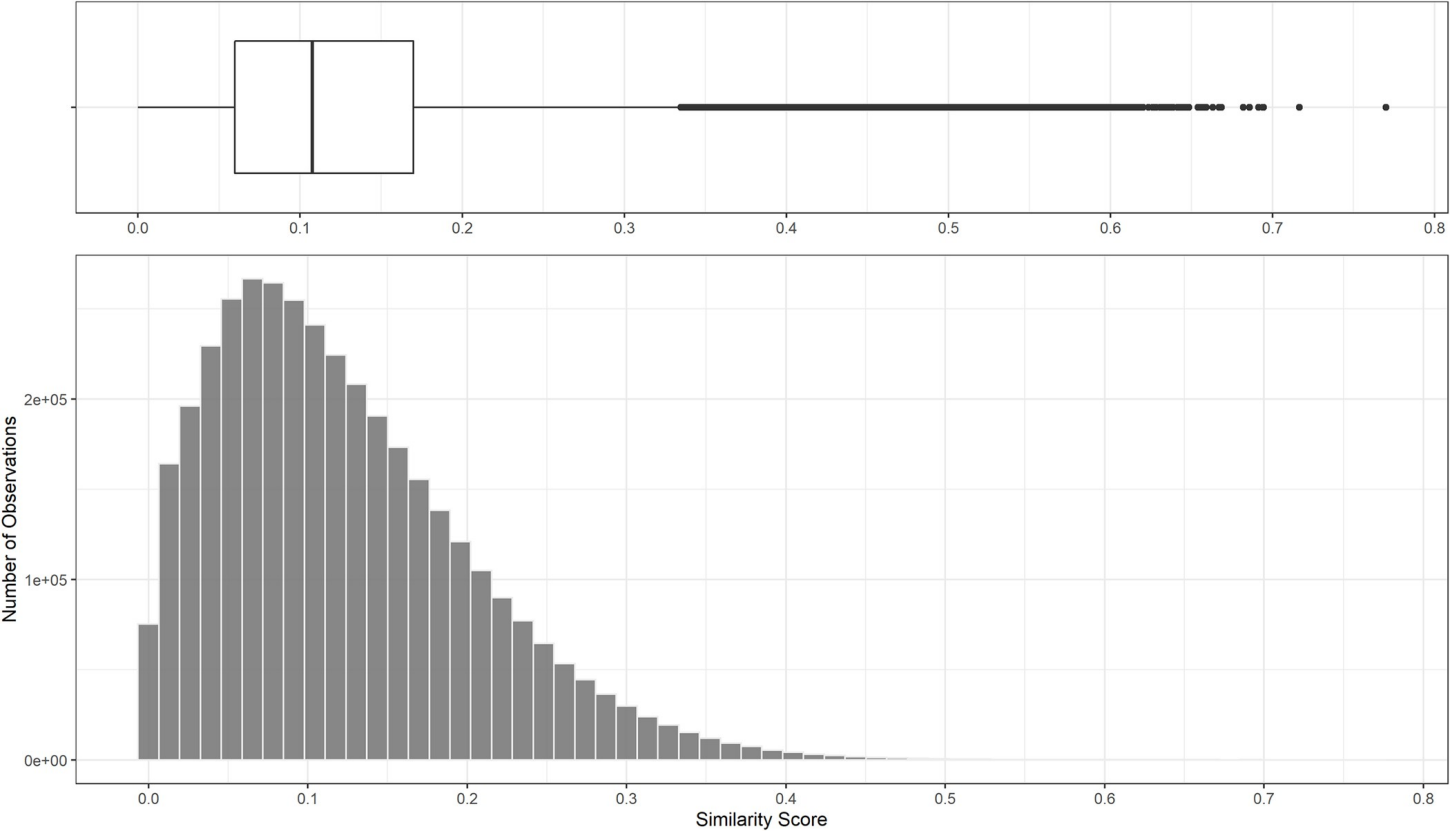
Histogram and boxplot of similarity score.

Then, we analysed the distribution of the two indicators to identify the thresholds for measuring the terminology’s standardisation. Figs 1 and 2 present the box plots and the histograms of each of the two indicators.

The *Percentage of Shared Skills* in Fig 1 does not exhibit any recognisable shape, presenting a peak around zero with an average value of 0.22%. Overall, the number of standard terms adopted in the job vacancies is low. Some outliers emerge in the box plot, where there is a set of points over the value of 20%, meaning that some ads share 20% of their content with the description of an ESCO occupation. Therefore, we selected this value as the threshold.

The *Similarity Score* in Fig 2 presents a right-skewed distribution with an average value of 0.12 and a maximum value of 0.76. So, for the most part, the job offers of ReliefWeb differ from the ESCO occupation (as we can see from the right part of the graph). This can be explained by the nature of ESCO, a general-purpose taxonomy that includes many jobs far from the scope of our analysis. In general, the similarity score is quite low, with an average value of 0.12 (0.11 median). There are some exceptions, as highlighted in the box plot, which register values of similarity higher than 0.30, proving that job vacancies somehow address the concepts described in the ESCO occupations taxonomy. By reading the pairs with a value of similarity in the range 0.3–0.4, we encountered many dissimilar pairs. As an example, the *Child Protection Senior Manager* from ReliefWeb and the *ICT Help Desk Manager* from ESCO are rated with a similarity score of 0.31: the first addresses a key role in leading project development for child protection services, the second organises the delivery of ICT services, ensuring the alignment with customers’ needs; both of them provide technical and managerial support for staff and partners, but in totally different contexts. Therefore, we selected a value of 0.4 as the acceptable similarity score threshold.

Combining the two thresholds allows us to identify four cases for the degree of standardisation of the NGOs’ job positions, as described in Table 4.

**Table 4:** Cases for the degree of standardisation of the NGOs’ job positions.

Case	*Percentage of* *Skills Shared*	*Similarity Score*	Number of pairs of a ReliefWeb job title and an ESCO occupation
**A**	High	High	12
**B**	Low	High	15,971
**C**	High	Low	191
**D**	Low	Low	3,752,528

We specify that a job offer from ReliefWeb can be associated with more than one ESCO occupation and, therefore, can fall in more than one case (on average, 2.08 cases for each job offer).

To confirm these observations quantitatively, we checked the statistical significance of the obtained results.

Firstly, the distributions of the two indicators significantly deviate from a normal distribution, as in Figs 1 and 2. The Shapiro-Wilk normality test [70] indeed rejected the null hypothesis of normal distributed data, with the Test Statistics W = 0.22711, p-value < 2.2e-16 for *Percentage of Shared Skills* and W = 0.93975 and p-value < 2.2e-16 for *Similarity Score*.

Secondly, we adopted a non-parametric test to verify whether the difference in the level of standardisation measured with the two indicators is statistically significant. The Wilcoxon test [71] can be used to compare means from samples that cannot be assumed to be normally distributed [72]. The Wilcoxon Signed-Ranks Test Statistic W = 682102301 with a p-value < 2.2e-16 for *Percentage of Shared Skills* and W = 5.998e+10, p-value < 2.2e-16 for *Similarity Score*. Therefore, we can reject the null hypothesis for both indicators, confirming the statistical significance of the result obtained and meaning that the means of high standardisation level in *Percentage of Shared Skills* (or in *Similarity Score*) is significantly different from the means of low standardisation level in similarity in *Percentage of Shared Skills* (or in *Similarity Score*). Thus, their combination can be used to derive guidelines towards improving the standardisation of job descriptions.

First, case A includes the pairs corresponding with ReliefWeb and ESCO, proving an alignment between the two sources. Case B encompasses the couples for which the job vacancy is not in line with the concepts in ESCO taxonomy, referring to the need for standardisation in the terminology adopted for Humanitarian recruitment service. Next, case C contains the pairs for which the occupation in ESCO is not in line with the definition of the job vacancy of ReliefWeb, suggesting the need for integration of new labels for jobs in ESCO. Finally, case D consists of the couples which generate different profiles. In the following sections, we analyse each case in depth.

### 4.3 Insights on jobs positions for emergency response

**Fig 3:**
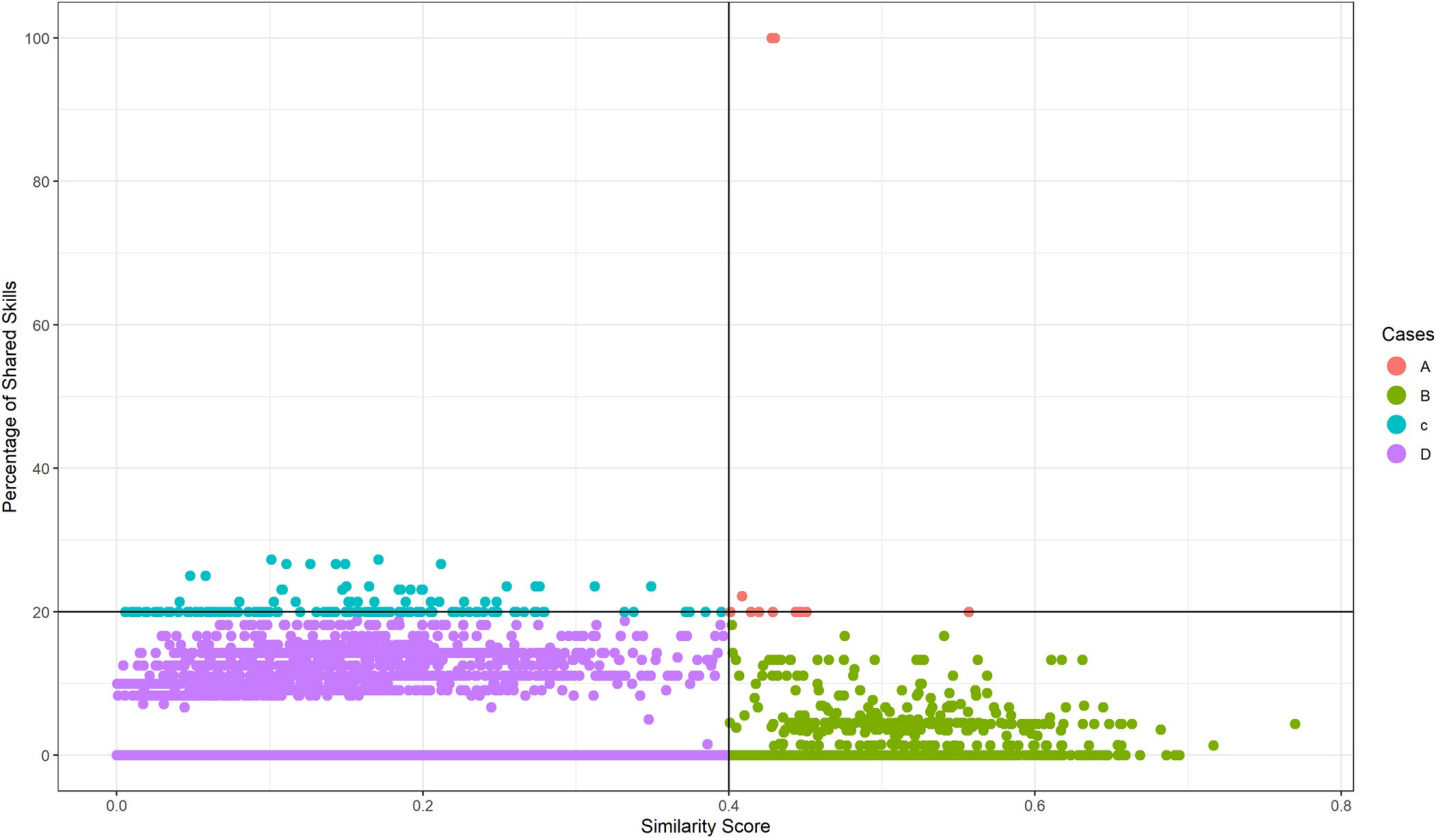
Cases’ map for the degree of standardisation of the NGOs’ job positions. The plot includes 2,819 pairs of pair job title Reliefweb and occupation ESCO (for which the percentage of skills shared and/or the similarity score were equal to the maximum value registered for each given pair). On the x-axis is reported the similarity score, on the y-axis is reported the percentage of shared skills.

We selected 2,819 pairs of pair job title ReliefWeb and occupation ESCO for which the P*ercentage of Skills Shared* and/or the *Similarity Score* were equal to the maximum value registered for each pair. In this sample, we still have all the 12 entries for case A. Then we obtained 1,296 pairs for case B, 148 for case C, and 22,024 for case D. In this sample, a given job offer from ReliefWeb can be associated on average to 19.28 ESCO occupations and 1.96 cases on average. Then, we map the four cases for the degree of terminology’s standardisation of those entries in a two-dimensional plot, reported in Fig 3, where the pair job title ReliefWeb and occupation ESCO are positioned based on their *Percentage of Shared Skills* measures and *Similarity Score*.

***Case A*: *Job vacancies in line with ESCO*.** Table 5 shows the 9 entries listed under this group. They relate to technical positions covering specific tasks in implementing Humanitarian interventions.

**Table 5:** Pairs of job titles from Reliefweb and occupations from ESCO are included in case A.

Job Title from ReliefWeb	Occupation from ESCO	Percentage of Shared Skills	Similarity Score
Clean Energy Specialists	Energy Consultant	100%	0,43
Senior Strategist, Finance Programme, Tara	Energy Consultant	100%	0,43
Senior Protection Information Manager Officer—Ethiopia	Data Entry Clerk	22,22%	0,41
Grants Analyst	Financial Analyst	20%	0,45
Country Representative DRC	Secretary General	20%	0,45
Administrative and Financial Coordinator	Financial Analyst	20%	0,56
SENIOR FINANCE AND GRANTS OFFICER	Financial Analyst	20%	0,45
Feed the Future Policy LINK–Accountant, Ethiopia	Financial Analyst	20%	0,42
NIGERIA–FINANCIAL COORDINATOR (H/F)–MAIDUGURI	Financial Analyst	20%	0,43
Global Programme Strategy and Innovation Director	Programme Funding Manager	20%	0,40
Global Programme Policy and Development Director	Programme Funding Manager	20%	0,41
Chief of Party	Financial Analyst	20%	0,44

By reading the list of extracted skills related to each pair, we can point out that they are very technical and distant from a typical profile of humanitarian workers, as defined in [4]. For example, some of the recurrent skills associated with the job ads in this category relate to *accounting*, *economics*, *and language skills*. Recurrent are also skills related to c*ommunication*, *management and planning*. Those are part of the general profile required to perform in the Humanitarian field, but they are irrelevant to the specific tasks. We can observe that applicants for these positions may often have a profit background and tend to have a short experience in the NGO environment, working for larger and more structured organisations, such as donors’ offices or UN Agencies. Therefore, this job offer appears to align with the ESCO language, referring to a more general demand in the labour market.

In this case, the results can be interpreted in the context of the Humanitarian Supply Chain as quantitative evidence of what has emerged from the literature analysis. In a fast-changing labour market, many positions in multiple industrial sectors and domains require transversal competencies addressing management, language skills, and communication. Therefore, those requirements are described with a well-established language, i.e., a less specific and more standard terminology, which means, in our analysis, closer to ESCO.

***Case B*: *Job vacancies not in line with ESCO*.** Despite the high similarity, the ReliefWeb positions in case B are not in line with ESCO classification, as they do not have many terms in common. Table 6 shows ten job profiles listed under this group. They related to technical positions covering very specific tasks in implementing Humanitarian interventions.

**Table 6:** Pairs of job title from Reliefweb and occupation from ESCO included in the case B.

Job Title from ReliefWeb	Occupation from ESCO	Percentage of Shared Skills	Similarity score
Project Coordinator	Programme Manager	4,35%	0.77
Project Management Officer	Programme Manager	4,35%	0.62
Project Manager	Programme Manager	4,34%	0.60
Grants Manager	Programme Manager	4,38%	0.59
Health & Nutrition Project Manager	Environmental Health inspector	6,67%	0.49
Disaster Risk Reduction Project Manager—Beirut—LEBANON	Secondary School Department Head	4,55%	0.48
Colombia—Health Project Manager in Tibú	Medical Laboratory Manager	5%	0.47
DDG Risk Education and Non-Technical Survey Project Managers—General Call	Rail Project Engineer	3,33%	0.46
EU Project Manager	strategic planning manager	0%	0.46
Chief of Party, Mozambique	Social Services Manager	0%	0.45

The entries of this case reported in Table 6 are all reflecting mid management positions in the humanitarian field. The high values of similarity prove that an alignment between Humanitarian recruitment and ESCO is possible. However, the low values for the *Percentage of Shared Skills* highlight the peculiarity of humanitarian workers, even when employed in apparently standard positions.

Managers in ESCO are described as responsible for the overall activities of enterprises, governments and other organisations. They formulate and review companies’ policies, laws, rules, and regulations; they are usually responsible for decision-making processes regarding the overall strategic and operational direction of a business or organisational unit, budgets, and selection and dismissal of staff. Field observations and the review of contracts released by International NGOs lead to note that it is not a necessary condition that managers have responsibility for all three of strategic and operational direction, budgets and staff selection and dismissal. The degree of autonomy they exercise may also vary. Indeed, some of the detected skills refer to the ability to lead processes in *health and safety*, *innovation*, *and emergency response*, which are specific to the Humanitarian working environment and difficult to translate into the general ESCO skills for the same positions. The critical difference is that supervisors are responsible only for the supervision of the activities of other workers, whereas managers have overall responsibility for the operations of an organisational unit. It can be noted that, *de facto*, what is implemented at a practical level in Humanitarian settings is very much in line with the ESCO taxonomy, but only at a practical level. Looking at the position of Disaster Risk Reduction Project Manager, as an example, the extracted competencies are defined as a mix of knowledge of topics—*English*, *French*, *geography*, *history*, and *topography—*and abilities to ensure data protection, manage and organise emergency response, as well as soft skills related to *communication* and *coordination*. This list makes sense for what the DRR Project Manager will conduct in the field during the assignment; the misalignment from ESCO, however, makes recruiting technical profiles with a Civil Protection background impossible, as other key skills are not present in the description. Moreover, a discrepancy between the information contained in the job vacancies and the contractual terms of reference of the profiles identified here emerges.

Therefore, this case proves the need to review the definition of requirements when preparing job vacancies for publications.

Thus, in the context of the Humanitarian Supply Chain, it is fundamental to balance generalization with specificity and to adapt the terminology when a more sectoral position is in demand. This result can suggest a possible expansion of the standard terminology, but also highlight the downside of high levels of standardization when it comes to convey peculiarities of the jobs.

***Case C*: *ESCO not in line with job vacancies*.** Case C shows limitations related to ESCO taxonomy. The analysis shows that the taxonomy does not envisage profiles with versatile knowledge, competence and skills in multiple management aspects, as it is required for the senior positions in the Humanitarian Sector. Table 7 illustrates ten different combinations in this category. This case presents another quantitative confirmation of the peculiarities of the jobs in the Humanitarian Supply Chain, which needs more specific terms to be properly described. Therefore, such results can indicate whether and at which level a standardisation of job descriptions is needed.

**Table 7:** Pairs of job title from ReliefWeb and occupation from ESCO included in the case C.

Job Title from ReliefWeb	Occupation from ESCO	Percentage of Shared Skills	Similarity score
MEL Director, Tanzania C3HP-HIV/TB	Political Scientist	27,27%	0.17
Re-advertisement: Investigation Assistant / Translator—National position/GOAL Syria in Amman	Proofreader	25%	0.05
Country Director	Investment Fund Management Assistant	23,53%	0.35
REACH Assessment Officer (Humanitarian Situation Monitoring) for South Sudan	Logistics Engineer	21,43%	0.15
CHILD PROTECTION EXPERTS—ICMC Deployment Scheme Roster	Behavioural Scientist	21,43%	0.04
ADVOCACY, CAMPAIGNS AND MEDIA MANAGER (INT7281)	Programme Funding Manager	20%	0.37
Grants Coordinator	Financial Analyst	20%	0.24
Senior Research Analyst, Decarbonisation and Net-Zero Transitions	Futures Trader	20%	0.14
Monitoring and Evaluation Lead	Criminologist	20%	0.10
ADVOCACY, CAMPAIGNS AND MEDIA MANAGER (INT7281)	Producer	20%	0.09

The entries of this case reported in Table 7 relate to Senior Management/Expert positions in the humanitarian field. The high values for the *Percentage of Shared Skills* suggest a proper definition of skills and knowledge relevant to the field. However, the low values of similarity prove that the definition of occupation in ESCO does not include figures that can be used in the humanitarian field.

Skills addressing technical qualifications such as *Apply Statistical Analysis Techniques; Government Policy; Government Policy Implementation* are relevant for the *MEAL Director* (MEAL is an acronym for Monitoring and Evaluation Lead). However, the algorithm associates this position with the ESCO occupation of Political Scientist without any relation to what the role of the latter will imply. A similar scenario is met for the *Investment Fund Management Assistant*, whose ESCO skills—*Analyse Economic Trends; Analyse Market Financial Trends; Assist in Fund Management; Banking Activities*, amongst others—have no link to the definition of what a *Country Director* in the Humanitarian field would do. The common skills are *Quantitative Data*, *Reporting*, *Data Analysis*, and *Design*.

***Case D*: *Different profiles*.** This case includes 3,752,547 combinations of jobs of ReliefWeb with ESCO occupations whose *Percentage of Shared Skills* measures and *Similarity Score* are low or equal to 0. Most of these entries do not provide any relevant insights, except to prove quantitatively the differences among two occupations (*Partnership Coordinator* from ReliefWeb and *Scrap Metal Operative* from ESCO). A part of this list includes positions that are typical of the sector and are not at all included in the ESCO repository. Some of them are now at the foundation of the criteria required in terms of response to emerging concerns within the sector, such as protection and gender.

IASC defines protection as “encompass(ing) all activities aimed at ensuring full respect for the rights of the individual in accordance with human rights law, international humanitarian law (which applies in situations of armed conflict) and refugee law” [73]. In the 2021 status of worldwide crises, such as Afghanistan, Ethiopia (Tigray), Belarus, to name a few, profiles related to developing and supervising interventions with expertise in this sector are paramount. It can be argued that for the delicate function these profiles encompass, the appropriate definition and standardisation of the skills and competence against which they are being evaluated becomes of utmost relevance to guarantee the appropriate accountability to the clients. The case of this position, usually addressed in the sector as *Protection officer/team leader*, can be commented on by referring to the job ads of *Protection Programme Development Manager for Honduras*. In ESCO, “world protection” is mentioned as a cross-sector skill, and the occupation related to it is *Human Rights Officer*. However, neither the similarity nor the shared skills are associated with the title and the occupation. Reading the definition of the *Human Rights Officer* in ESCO, the question arising is on the specifications in the ReliefWeb job vacancies because, if properly formulated, the Protection Programme Development Manager would have a higher number of shared skills and most probably fall in case C. As seen above, the restructuring and grouping of occupations would be envisaged for ESCO.

Positions associated with Gender equality, Prevention of Gender-Based Violence and discrimination have become paramount in recent years [1,2]. The Gendermarker and related audit is a requirement of all major European donors, asking for full transparency and a dedicated policy to demonstrate that no gender-based bias applies in the organisation. Because of scandals involving major organisations in the last five years, donors also require a PSEA—*Protection from Sexual Exploitation and Abuse*—procedure to be in place—specific positions to draft, implement, and audit are required under each grant/operation. The position of *Senior Gender Advisors GenCap* from ReliefWeb aligns with the Baggage Flow Supervisor position in ESCO. Gender is listed in ESCO as a skill associated with sociology and cultural studies. No occupation in ESCO contains the term “gender”. On the contrary, in the Humanitarian sector, Gender Advisers cover an extremely prominent role in the design and correct implementation of projects. So, ESCO could need to revise the definition of the occupation to include gender-related roles.

The conclusion that can be drawn from the analysis of this case is that ESCO could enlarge the spectrum of occupations to include the ones pertinent to the humanitarian sector and that the definition published in ReliefWeb may contain terms less sector-specific and more in line with other sectors. This result can be interpreted as the emergence of new terminology from a specific domain/sector, in this study, the Humanitarian Supply Chain, for the standard taxonomy of competencies, in this study ESCO. Indeed, the new needs of the labour market constantly feed the taxonomy to ensure an up-to-date language for employers and employees.

### 4.4 Summary

In Section 1, we formulated our Research Question (Does the European Humanitarian HR supply chain use standard terminology in its HRM processes?). Here, we want to summarise the achieved answers.

The quantitative approach to analysing HR practices and performance in humanitarian operations exploited job vacancies from ReliefWeb and the European Classification of Skills and Occupations (ESCO). We assessed the terminology adopted in addressing skills and jobs in the humanitarian sector using text mining techniques, measured the level of standardisation of the positions, and tested the statistical relevance of the results.

Using ESCO as a central reference for the terminology to define key profiles, it is clear that Humanitarian HR is not highly standardised, nor is ESCO prepared for the moment to include profiles related to the humanitarian sector. This is proven both by the *lacunae* in the job vacancies on ReliefWeb, confirmed by cases B and D, and the ones of ESCO, as proven in Case C. However, it can be argued that there is room for improving the alignment of the humanitarian job vacancies to ESCO, as demonstrated in case A.

The research showed that an appropriate taxonomy can be linked to evaluation grids for each competence and, therefore, can ease the selection process. A different evaluation system could serve as a standard system of verification of progress and results in the field. Therefore, this would lead to a more competent offer in the job market because competencies will be audited against objective standards. A higher competence level contributes to increased accountability towards clients and donors; consequently, the degree of acceptance of international humanitarian support is ameliorated. In the increasingly volatile environment in which the humanitarian sector operates, the perception of the appropriate use of donors’ resources and the respect of the guiding principles of humanitarian actions are paramount not only for a more transparent use of funding but also not less importantly, for an increased safety for the operators. Higher performance standards of the operators are necessary to increase the confidence and trust amongst the population receiving the interventions. Authors in [74], in analysing the relationship between accountability and management in humanitarian organisations, on the contrary, take a stand against t*he new humanitarian management*; for the authors, the excess of standardisation and normative control leads to an impoverishment of the social values necessary for potential NGO workers. Cazenave and Morales build on the review proposed in [75], stating that the downward and hierarchical approach to NGO staff management has further reduced the accountability towards the population, being donors and stakeholders oriented only. In front of such an open debate, the use of text mining proposed in this paper showed that it is possible to propose a mathematical model to quantify the elements of standardization, whether they approved or argued against.

The model measures the alignment among the analysed sources and sheds light on the skills’ needs in the Humanitarian sector. Equally, we can argue that this kind of approach could be effective in assessing the coverage of ESCO’s concepts in relation to the Humanitarian sector. Restructuring the definition of the job vacancies in ReliefWeb and similar sites can be the first step towards alignment with the ESCO taxonomy and, therefore, the standardisation of the definitions of the skills and competencies. As seen under the results’ analysis, prior to achieving this, both ESCO and the Humanitarian Sector need to take further steps toward a more precise definition of their taxonomy.

The hypothesis of this research considered the longer-term outcome of the possible creation of an auditable selection process for operators in the humanitarian sector based on the standardisation of skills. NGOs are subject to continuous auditing and evaluation against Key Performance Indicators (KPIs) at the organisational level; to maintain access to funding, the organisations need to prove regularly their compliance with a long list of international standards, which different donors repeat at different periods of the year. Additionally, NGOs are audited at the project level, both during operations and after the final financial report stage. However, it is not common practice that such an audit conducts an evaluation of the performance of the individual staff members working at the field and headquarters levels. May the alignment with ESCO become a standard practice, individuals in the organisations can be held responsible for the failure to deliver to the clients, and not only at the organisational and managerial levels.

## 5. Conclusion

The application of quantitative data extraction, analysed in this study, gives a scientific and unbiased view of the required standards and characteristics of the least rigorously defined profile, the humanitarian worker. Whereas access to people in need is shrinking and aid is more and more politicised, the role of humanitarian workers becomes central; the standardisation of skills and competencies requested in job vacancies could support the selection of human resources to guarantee the appropriate delivery of aid. The humanitarian sector needs to rethink the key skills and competence to make them measurable and relevant.

To conclude, we must acknowledge some limitations of our work. First, for what concerns the methodology, we used only string-matching algorithms to extract skills from job vacancies, i.e., the gazetteer-based Named Entity Recognition method [76], as we used ESCO as a list of terms to be detected in the unstructured text of job vacancies. Further techniques can be applied for this purpose, such as a rules-based approach (regular expressions and pattern; [77]) and machine learning algorithms [78], which can lead to higher-quality results. In addition, we adopted only the European Classification ESCO to detect skills. However, other taxonomies can be added, such as the US O*NET information portal (https://www.onetonline.org/), to increase the recall of the results. Secondly, the scope of this work is limited to the job positions available in the humanitarian sector for Non-Governmental Organizations (NGOs) with a legal base in Europe and recruitment from the United Nations (UN) is not considered. This limitation of scope is due mainly to the peculiarities of UN recruitment criteria and job advertising, which are not generally part of ReliefWeb, and we wanted to leverage the open access data of this portal, given their availability and the possibility to replicate the approach. Future studies may, therefore, expand both the methodological approach and the scope of the research.
